# The causal impact of childhood obesity on bone mineral density and fracture in adulthood: A two-sample Mendelian randomization study

**DOI:** 10.3389/fnut.2022.945125

**Published:** 2022-09-14

**Authors:** Yuehui Liang, Ming-Gang Deng, Qinghong Jian, Minjie Zhang, Shuai Chen

**Affiliations:** ^1^School of Public Health, Wuhan University, Wuhan, China; ^2^Da Ping Hospital, Third Military Medical University, Chongqing, China

**Keywords:** fracture, bone mineral density, causal inference, obesity, Mendelian randomization

## Abstract

Observational studies have indicated the associations between obesity with bone mineral density (BMD) and fracture but yield inconsistent results. The impact of childhood obesity on bone health in adulthood is even less clear. The present study adopted the Mendelian randomization methods to determine whether the genetically predicted childhood obesity was causally associated with BMD and the risk of fracture. Genetic variants were extracted from genome-wide association studies (GWAS) to identify childhood obesity loci [IEU open GWAS project: childhood obesity (ID: ieu-a-1096)] and single nucleotide polymorphisms (SNPs) as instrumental variables to investigate causality. We used two-sample univariable Mendelian randomization (MR) to estimate causal relationships between childhood obesity on BMD and fracture subtypes based on SNPs from European samples. To avoid bias, Cochran's *Q* test and leave-one-out variant analysis were performed. The MR analysis shows strong evidence that childhood obesity is causally associated with eBMD (OR 1.068, 95% CI 1.043–1.095, *P* < 0.001) and a weak decreased risk of leg fracture (OR 0.9990, 95% CI 0.9981–0.9999, *P* =0.033) based on the inverse variance weighting (IVW) method. After adjusting for diabetes and adult obesity, the results of eBMD remained the same. The MR analysis revealed sufficient evidence to indicate childhood obesity was causally associated with increased BMD and decreased risk of leg fracture in adults. Childhood obesity could be taken into consideration when assessing eBMD.

## Introduction

Epidemic obesity in childhood has emerged as a huge nutritional burden worldwide over the past few decades (1). Approximately 40 million children under 5 years of age presented overweight in 2018 (2) and 18% of youth aged 5–19 globally were overweight or obese in 2016 according to Global Health Observatory data (3). The high prevalence of childhood obesity is linked to a cascade of metabolic comorbidities in later life, such as diabetes mellitus and cardiovascular diseases, which are most likely to increase morbidity and decrease quality of life (4). Bone health, despite being little addressed, is closely related to the metabolic level of the body and cannot be overlooked.

Worldwide, 21 million men and 137 million women are estimated to have a fracture history every year (5). Globally, there were 178 million new fractures in 2019 (an increase of 33.4% since 1990), and 455 million cases of acute or long-term symptoms of prevalent fractures (6). In the UK, the annual incidence of fractures is estimated at 3.6%, with a lifetime prevalence of fractures approaching 40% (7). Hip fractures and other types of vulnerable fractures not only have a far-reaching impact on the quality of life but also bring a heavy economic burden to society. Only one-third of these fracture patients can recover their function (8). Study has reported a 4-fold increase in mortality in women and a 7-fold increase in mortality in men in the first year after hip fractures (9).

Bone mineral density (BMD) is an important determinant of the risk of hip fracture. In recent years, growing evidence indicated a significant association between childhood obesity and bone health. Previous studies have found that childhood and adolescence with obesity have increased bone mineral content and BMD (10). On the contrary, it is reported that obesity places children at higher risk for supracondylar humerus fractures than non-obese children (11). In addition, obese individuals, especially those elderly people, have a negative association between body fat and BMD (12). Obesity is not conducive to the bone mineral density of children aged 0–5 years in China (13). The mechanism and factors that affect bone density appear to be especially complex. In regards to the influence of obesity on bone health, findings are controversial. The paradoxical mechanism behind obesity and bone metabolism may be attributed to differences in genetic background differences and a proxy for unmeasured lifestyle confounding factors or conjoint health exposure factors (14). Obesity is a complex disease involving multiple genes and processes. Some influential researchers have established a connection between obesity and gene single nucleotide polymorphisms (SNPs) (15–17).

Mendelian randomization (MR) is an instrumental variable analysis method based on genetic variants associated with the risk factors of interest that serve as un-confounded markers of exposure factors, which has been applied in genetic epidemiology to explore whether the association of co-occurrence traits reflects causality or simple correlation (18). Genetic variants divide the study population into subgroups that are similar to treatment groups in a randomized controlled trial, they are not associated with the outcome through any pathway and differ systematically *via* the exposure of interest (known as SNPs), but not concerning confounders. If instrumental variable assumptions are all met, the association between genetic variation and results means that the risk factors of interest have a causal impact on the results (19).

Despite the apparent simplicity, the association between obesity and BMD, the fracture is not straightforward. Hence, we will evaluate the impact of childhood obesity on bone mineral density and fracture in adulthood, and if so, to what extent. In this study, we used genetic variants associated with childhood obesity as un-confounded instruments to investigate the causality between this exposure and BMD and fracture (individuals of similar European origin in cases and controls). To disentangle the causal link behind these associations, we applied two-sample MR to combine summary statistics on the genetic variant to exposure and outcome associations from large samples and provide indicators of the strength of the association between exposure and outcome.

## Materials and methods

### Data sources

This two-sample MR study adopted summary-level genetic data derived from The MRC IEU OpenGWAS data infrastructure. The name of this dataset is IEU open GWAS project: childhood obesity (ID: ieu-a-1096). It consisted of 5,530 cases (≥95% BMI reached before the age of 18 years) and 8,318 controls, 2,442,739 variants (18). All reported genomic coordinates were in HG19/GRCh37.

The summary-level data on heel BMD (eBMD) were accessed from a GWAS meta-analysis using European samples (20). The femoral neck (FN), lumbar spine (LS), and forearm (FA) are the three common skeletal sites of postmenopausal women and men for measurement of BMD based on DXA obtained from the GWAS conducted by Zheng et al. (21). Heel bone mineral density (eBMD) was assessed in 426,824 participants by quantitative ultrasound speed of sound (SOS) and broadband ultrasound attenuation (BUA) by the Sahara Clinical Bone Sonometer (Hologic Corporation, Bedford, MA, USA) (22). The heel quantitative ultrasound can be applied to measure BMD, and its degree is similar to that of dual-energy X-ray absorption. The method is a cheap, easy to implement, and radiation-free technology and is generally suitable for large-scale population screening (23). All the participants with age across the cohorts between 39 and 75.8 years and a sex ratio of 1.2 (female to male). The population characteristics were male 39.0–75.8 {Mean [±standard deviation (SD)] age was 59.0 ± 8.1} years old and female 39.0–74.1 (59.0 ± 8.1) years old. Mean ± SD BMD levels were 0.56 ± 0.12 g/cm^2^ in men and 0.51 ± 0.11 g/ cm^2^ in women.

To maximize the statistical ability to detect gene loci, we selected two common fracture sites, leg (2,988 fracture cases and 457,352 controls) and spine (1,036 fracture cases and 459,304 controls) confirmed by medical and radiological reports, which were successfully used to test BMD variation associated with a fracture in previous studies (18). Our analysis in the study was originated from publicly available genome-wide association study (GWAS) summary statistics without additional ethical approval and informed consent.

### Instrumental variable selection

For the causal interpretation of MR analysis to be effective, three key assumptions must be met (Supplementary Figure 1).

**Figure 1:**
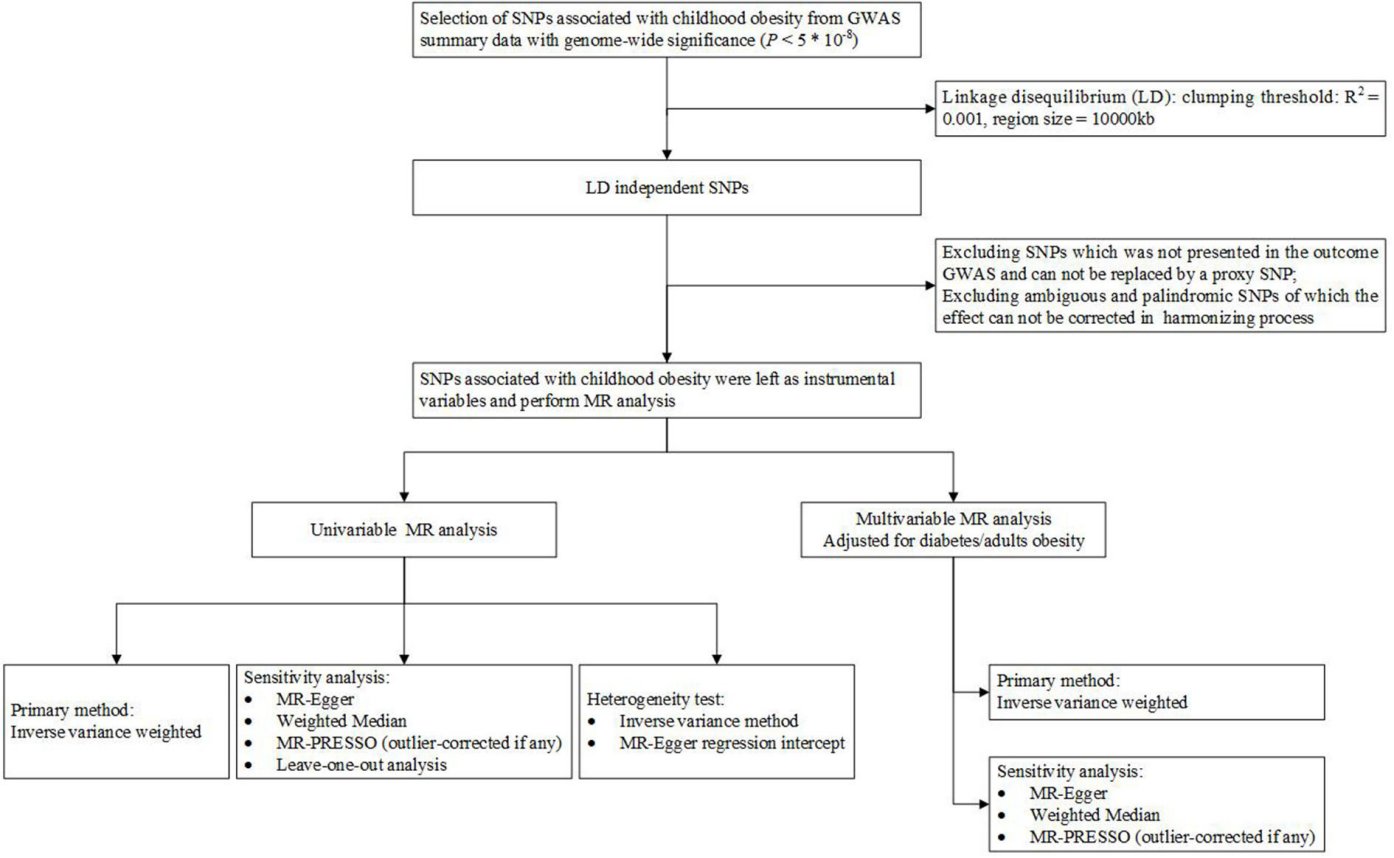
Flowchart of this study.

In short, (1) these genetic variations should be robustly related to childhood obesity, (2) not associated with any confounding factors of childhood obesity, bone mineral density, and fracture, and (3) childhood obesity only by affecting BMD and fracture (24). We selected SNPs associated with childhood obesity from GWAS summary statistics. The SNPs associated with childhood obesity were extracted as instrumental variables at the genome-wide significant level (*P* < 5 × 10^−8^) from the meta-analysis of GWASs encompassing 13,848 European-descent individuals. We identified 5 SNPs associated with childhood obesity at genome-wide significance (*P* < 5 × 10^−8^) (Supplementary Table 1) as the primary genetic instruments for exposure. The genome-wide association test was adjusted for age, gender, study location, genetic principal components, relatedness, and other study-specific characteristics. All the SNPs used were independent and were not in linkage disequilibrium when the distance exceeded 10,000 kb and R^2^ ≤ 0.001. The research flowchart of this study is shown in Figure 1.

### Statistical analysis

The Wald method was used to derive the ratio of the SNP-outcome estimate to the SNP-exposure estimate. We applied two-sample MR to obtain the potential causality between childhood obesity on bone mineral density and fracture, and combined the ratio estimates for each used SNP for one trait by using multiplicative random effects, including three MR methods: inverse variance weighting (IVW), weighted median (WM), and MR Egger (25–27).

The IVW method could satisfy the most precise estimates but could be affected by invalid instrumental variables and pleiotropic effects. Thus, for bone mineral density and fracture as well as associations reaching the conventional significance level (*P* < 0.05) (25), we further conducted two sensitivity analyses based on the weighted median and MR-Egger methods to give an unbiased estimate and adjust for possible pleiotropy (27). The weighted median method orders the MR estimates of each instrumental variable weighted for the reciprocal of its variance if at least 50% of the instrumental variables are effective. The MR-Egger regression gave an unbiased estimate by detecting and adjusting for pleiotropy albeit if low power is present (26). The MR-PRESSO test reduced the effects of heterogeneity and level of pleiotropy by removing outliers (28). We subsequently conducted a multivariable MR analysis to adjust the general risk factors that were genetically correlated with BMD. We used publicly available summarized data regarding the genetic association of instruments with BMD from MRC-IEU (426,824 individuals).

Cochrane Q value was performed to evaluate the heterogeneity between estimates of SNPs in each analysis. In addition, we investigated the PhenoScanner database (a tool for human genotype-phenotype associations) to assess possible pleiotropy for the SNPs associated with childhood obesity. All the analyses were two-sided and applied in R software (version 4.0.1).

## Results

We included some SNPs as instrumental variables of childhood obesity in this MR analysis in the risk of BMD and fracture (Supplementary Table 1). The results between childhood obesity and BMD and fracture obtained from the MR analysis were shown in Table 1 and Supplementary Figure 2.

**Table 1:** Univariable MR results for childhood obesity on eBMD and leg fracture risk.

	**eBMD**			**Leg fracture**		
	**OR**	**95%CI**	***P***	**OR**	**95%CI**	***P***
IVW	1.068	1.043, 1.095	<0.001	0.9990	0.9981, 0.9999	0.033
Weighted median	1.067	1.048, 1.086	<0.001	0.9988	0.99775, 0.99997	0.045
MR Egger	1.133	0.927, 1.386	0.310	0.9940	0.9880, 1.000	0.175
MR-PRESSO	1.067	1.046, 1.089	0.002	0.9993	0.9982,1.0004	0.306
MR-PRESSO global test			0.011			0.107
Cochran Q			<0.001			0.414
MR-egger pleiotropy			0.602			0.239

IVW, inverse-variance weighted; MR, Mendelian randomization.

**Figure 2:**
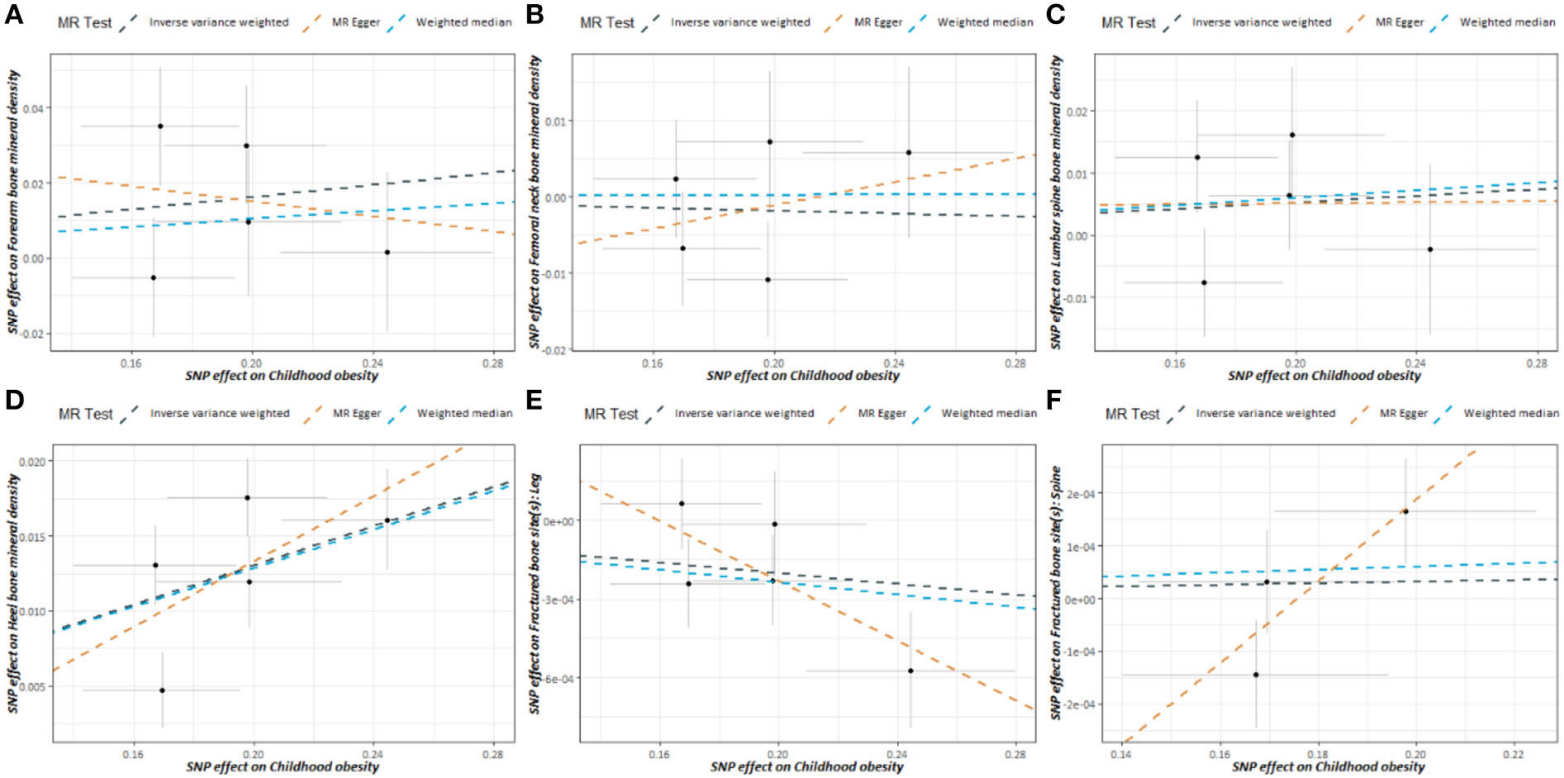
Scatter plot of childhood obesity-associated SNPs potential impacts on BMD and the risk of fracture in adulthood. **(A)** Forearm BMD, **(B)** Femoral Neck BMD, **(C)** Lumber Spine BMD, **(D)** eBMD, **(E)** leg fracture, **(F)** Spine fracture. The black dots represent the association of individual IV with childhood obesity and the association of individual IV with BMD, fracture. Vertical and horizontal lines denote the 95% CI of OR for each IV. The slope of each line denotes the estimated causality per MR method. BMD, Bone mineral density; IV, instrumental variable; MR, Mendelian randomization.

The slope of each line denotes the estimated causality per MR method in Figure 2. The slope of eBMD was positive, but the slope of leg fracture was negative. Genetical variants predicted childhood obesity was positively associated with BMD and inversely associated with leg fracture in adulthood.

The leave-one-out sensitivity analysis suggested that MR analysis results were a weak association between childhood obesity and the risk of leg fracture (Supplementary Figure 3). The MR-Egger regression indicated that there was no evidence of strong horizontal pleiotropy between IVs and outcome, indicating that the horizontal pleiotropy was balanced (*P* > 0.05).

In multivariable MR analyses with other variables from the UK Biobank (https://www.ukbiobank.ac.uk/), the effect of childhood obesity on eBMD remained after adjusting for diabetes and obesity in adulthood (Table 2). Likewise, the effect of childhood obesity on leg fracture in adulthood after adjustment for diabetes in adulthood remained, although this direct effect was not significant after adjusting for adult obesity.

**Table 2:** Multivariable MR results for childhood obesity on eBMD and leg fracture risk.

	**eBMD**			**Leg fracture**		
	**β**	**Std. error**	***P***	**β**	**Std. error**	***P***
**IVW**						
a	0.040	0.016	0.012	−0.001	0.0004	0.027
b	0.078	0.031	0.012	−0.001	0.001	0.033
**Weighted median**						
a	0.057	0.015	<0.001	−0.001	0.001	0.110
b	0.077	0.029	0.008	<0.001	0.001	0.730
**MR Egger**						
a	0.072	0.020	<0.001	−0.001	0.001	0.018
b	0.083	0.054	0.122	−0.001	0.001	0.523
**MR-PRESSO**						
a	0.040	0.016	0.016	−0.0009	0.0004	0.032
b	0.078	0.031	0.054	−0.0006	0.0008	0.489
**MR-PRESSO global test**						
a			<0.001			0.330
b			0.003			0.316
**Cochran Q**						
a			<0.001			0.307
b			<0.001			0.361

Multivariable 1 adjusted for diabetes in adulthood.

Multivariable 2 adjusted for obesity in adulthood.

The result of the IVW method suggested that genetically predicted childhood obesity was positively associated with the eBMD (OR = 1.068, 95% CI = 1.043–1.095, *P* < 0.001), and the result of MR PRESSO analyses (OR = 1.068, 95% CI = 1.061–1.075, *P* < 0.001) and WM method (OR = 1.067, 95% CI = 1.048~1.085, *P* < 0.001) remained consistent with the result of the IVW analyses. Although the MR-Egger method showed wider CIs due to lower statistical power, the recommended effect direction was the same (OR = 1.133, 95% CI = 0.927–1.386, *P*=0.31). However, no causal effect of childhood obesity on FA-BMD, or LS-BMD, or FA-BMD was found in this part. Then, we calculated the individual and pooled MR estimates. In the association between childhood obesity and fracture, the IVW method ORs of leg fracture was 0.9990 (95% CI 0.9981–0.9999; *P* = 0.033) per genetically predicted when children were obese or not. The OR estimates were less precise in MR Egger, though the direction was the same. As for fracture outcome, Cochran's *Q* test suggested that the effect estimates across all instrumental variables were not heterogenous (*Q*_Egger_ = 2.860, *p*_Egger_ = 0.414, *Q*_IVW_ = 5.014, *p*_IVW_ = 0.286, Supplementary Figure 4).

## Discussion

Studies on childhood obesity and bone health in adulthood are controversial. To the best of our knowledge, the present study is the first MR study that systematically investigated the causality of genetic liability to childhood obesity was positively associated with bone mineral density, whereas genetically predicted childhood obesity was inversely associated with fracture. Our findings have significant implications for the precise evaluation that childhood obesity could be considered as a signal that indicates wellbeing BMD in adulthood. Our study reconfirms that bone growth trajectories are determined early in life. This report strengthened the evidence of causality between childhood obesity and BMD and fracture. Moreover, our findings may provide important information for the precise implication of fracture and bone mineral density in youth with obesity. This study highlights the beneficial impact of obesity in childhood on BMD for some specific periods.

In this study, we screened the SNPs with the genome-wide association and independent inheritance as instrumental variables to examine the causal association between exposure and outcomes. Moreover, a representative sample of European-origin participants was first selected to explore the bone health of adults from obesity in childhood. The results are also based on the risk of developing fracture, a disease of decreased bone density, providing clinically valuable epidemiological evidence in this field. The associations between childhood obesity and leg fracture were established. But we should reveal this association with more caution and further research, possibly due to the limited significance of the clinical practice.

According to observations, the impact of childhood obesity on the BMD and risk of fracture was not always consistent, potentially due to the confounding of undetermined sources. The MR was an effective tool for identifying the causal association between potential exposure and disease while circumventing confounders, which might be the main cause of these inconsistent results. In previous studies, obesity in childhood was often interpreted to increase the risk of chronic diseases in adulthood. However, there is also strong evidence suggesting that bone mineral content is higher in obese children than in normal-weight peers. The potential mechanisms by which viewpoint could be body adiposity representing a mechanical load, which was beneficial for bone strength. Some influential biological mechanisms could be explained as bone marrow adipocytes share many of the characteristics of extramedullary adipocytes (i.e., adipocytes present in adipose tissue) (29). Thus, factors that modulated metabolic function and sealed cell fate in peripheral fat depots might also affect bone marrow adipocytes. Furthermore, bone marrow adipocytes were an important source of paracrine factors that play an active role in the bone marrow (23). Therefore, bone marrow adipocytes might directly secrete bone-regulating adipokines, such as leptin and adiponectin, into the local microenvironment, thereby affecting osteoblast function and osteoblast abundance (30). Hu et al. identified several loci with pleiotropic effects in patients with fracture and obesity, which suggested an underlying genetically determined mechanism for fracture and obesity (31). Therefore, it could be inferred that childhood obesity would cause decreased risk of fracture incidents in adulthood even at the level of normal bone mineral density. The previous study has detected a positive association between type 2 diabetes and hip bone mineral density (24). We found similar results in our MR study, but the effect on BMD may be site-specific. Fat-related metabolism in different types of bone was significantly different (32). The instrumental variables used in our MR study were mainly located in the genes TNNI3K (rs1040070), TMEM-18 (rs4854344), MC4R (rs571312), FAIM2 (rs7138803), and FTO (rs9941349). All these genes are strongly associated with body mass index and obesity risk, related mechanisms involving regulation of cell migration, and the defects in MC4R were a cause of autosomal dominant obesity. The above mechanisms suggested by instrumental variables related genes might confer genetic insights into the causality between childhood obesity and bone mineral density and fracture. The sensitivity of bone to fracture is inversely proportional to the strength of the bone and proportional to the force applied. Bone strength also increases with BMD (33). Apparently, the role of weight gain in obese individuals may be more pronounced in supporting bones (e.g., vertebral bodies or foot bones), possibly due to moderate mechanical stress stimulation that favors bone formation. From the clinical point of view, increased bone density has a certain positive protective significance for the prevention of leg fracture, but not for spine fracture.

## Strengths and limitations

Our study has several strengths, including causal inference, standardized approach to BMD and fracture measurement, and a comprehensive assessment of covariates. However, our study had several limitations worth mentioning. First, observational studies have found that different definitions of obesity (body fat or BMI) may have different associations with BMD (33, 34). This study did not do the specific analysis of the relevant aspects. We look forward to follow-up research reports. Second, though we have applied phenoscanner to screen potential confounding factors, it was difficult to verify the independence of instrumental variables and confounding factors affecting the exposure and outcome relationship (independence hypothesis) and that genetic variation could only affect the outcome through exposure factors, but could not affect the outcome through other ways (exclusive hypothesis). This process might also introduce potential bias, such as physical activity. Third, the public source did not provide the relevant individual demographic or clinical information, such as the other indicator for BMD or fracture, and the status of childhood obesity were unknown or unobserved, which might impair the clinical significance of our study.

## Conclusion

From the study of over 10,000 participants, we strengthened the evidence that the main genetic determinants of childhood obesity also influence BMD and leg fracture in adulthood, which is notably in favor of causal associations of childhood obesity with BMD and fracture. Our study reconfirmed that bone growth trajectories are determined early in life, among the potential risk factors we evaluated. More advanced methods by the gold standard (dual-energy X-ray absorptiometry, DXA) and updated MR analysis were urgently needed to confirm our results and obtain less bias estimation and better accuracy or GWAS summary data. Foremost, our results suggested a lower index of suspicion for fracture, and reinforcement of BMD screening recommendations among patients with obesity in childhood did not have to enable the early detection of BMD. This study highlights the beneficial impact of obesity in childhood on bone health for some specific period.

## Data availability statement

The data that support the finding of this work was available in the UK Biobank at https://www.ukbiobank.ac.uk/. These data were derived from the IEU OpenGWAS Project at https://gwas.mrcieu.ac.uk/, last accessed 21 January 2022.

## Ethics statement

Ethical approval was not provided for this study on human participants because we used a public database and no additional ethical approval was required. The patients/participants provided their written informed consent to participate in this study.

## Author contributions

Conceptualization: SC. Formal analysis: M-GD and YL. Writing—original draft preparation: YL. Writing—review and editing: QJ, MZ, and SC. All authors contributed to the article and approved the submitted version.

## Funding

This work was supported by the Fundamental Research Funds for the Central Universities (2042021kf0042) and the fund of the Beijing Engineering and Technology Research Center of Food Additives, Beijing Technology and Business University (BTBU).

## Conflict of interest

The authors declare that the research was conducted in the absence of any commercial or financial relationships that could be construed as a potential conflict of interest.

## Publisher's note

All claims expressed in this article are solely those of the authors and do not necessarily represent those of their affiliated organizations, or those of the publisher, the editors and the reviewers. Any product that may be evaluated in this article, or claim that may be made by its manufacturer, is not guaranteed or endorsed by the publisher.
