# Supplementary material for: The causal impact of childhood obesity on bone mineral density and fracture in adulthood: A two-sample Mendelian randomization study
Source: Front Nutr. 2022 Sep 14;9:945125. doi: 10.3389/fnut.2022.945125 (PMC9515586; doi:10.3389/fnut.2022.945125)
Supplement: Supplementary file 1 [file Data_Sheet_1.zip › Supplementary Figures.docx]

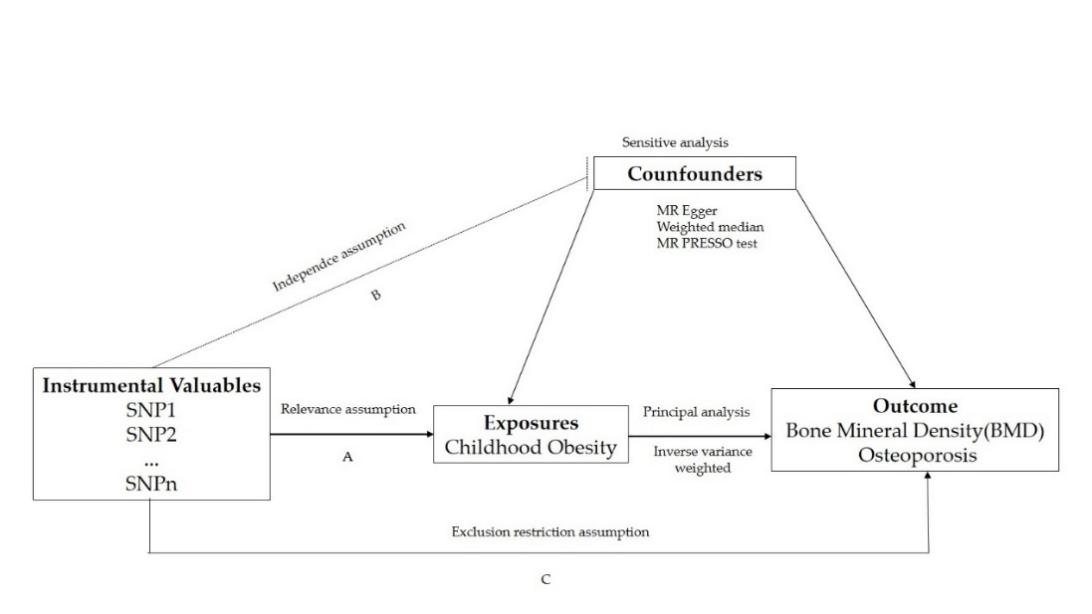


**Supplementary Figure 1**. Schematic of the MR study and key assumptions. A, Instrumental variables associate with the risk factor of interest. B, Instrumental variables share no common cause with the outcome. C, Instrumental variables do not affect the outcome except through the exposure. SNP, single nucleotide polymorphism.

**
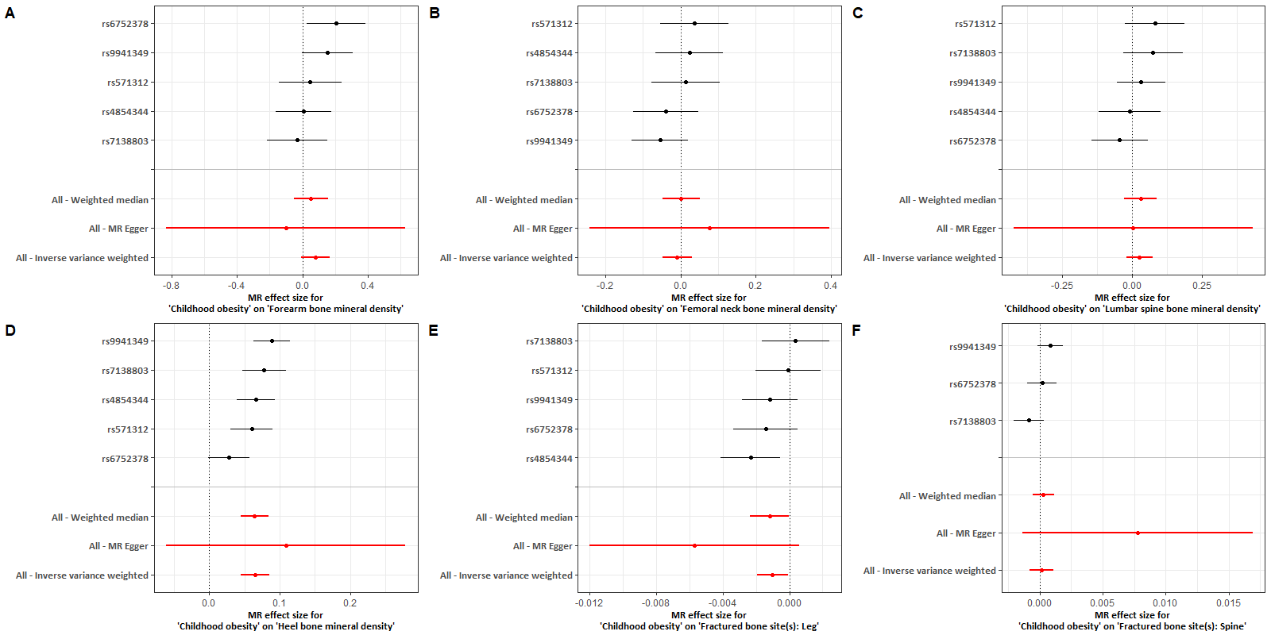
**

**Supplementary Figure 2**. Forest plot of childhood obesity associated SNPs potential impacts on BMD and fracture in adulthood. (A) Forearm BMD, (B) Femoral Neck BMD, (C) Lumber Spine BMD, (D) eBMD, (E) Leg fracture, (F) Spine fracture. BMD: Bone mineral density; MR: Mendelian randomization.


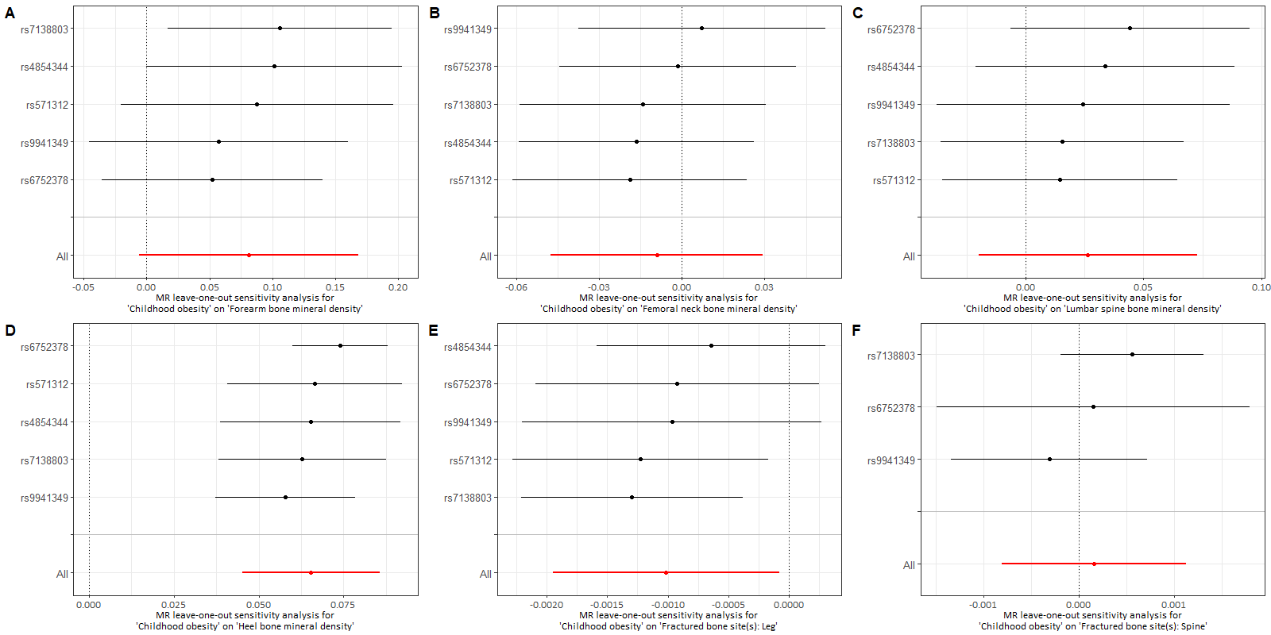


**Supplementary Figure 3**. Sensitivity analyses for the causal association of childhood obesity and the impact on BMD and the risk of fracture in adulthood using the leave-one-out approach. (A) Forearm BMD, (B) Femoral Neck BMD, (C) Lumber Spine BMD, (D) eBMD, (E) Spine fracture, (F) Leg fracture. BMD: Bone mineral density; MR: Mendelian randomization.


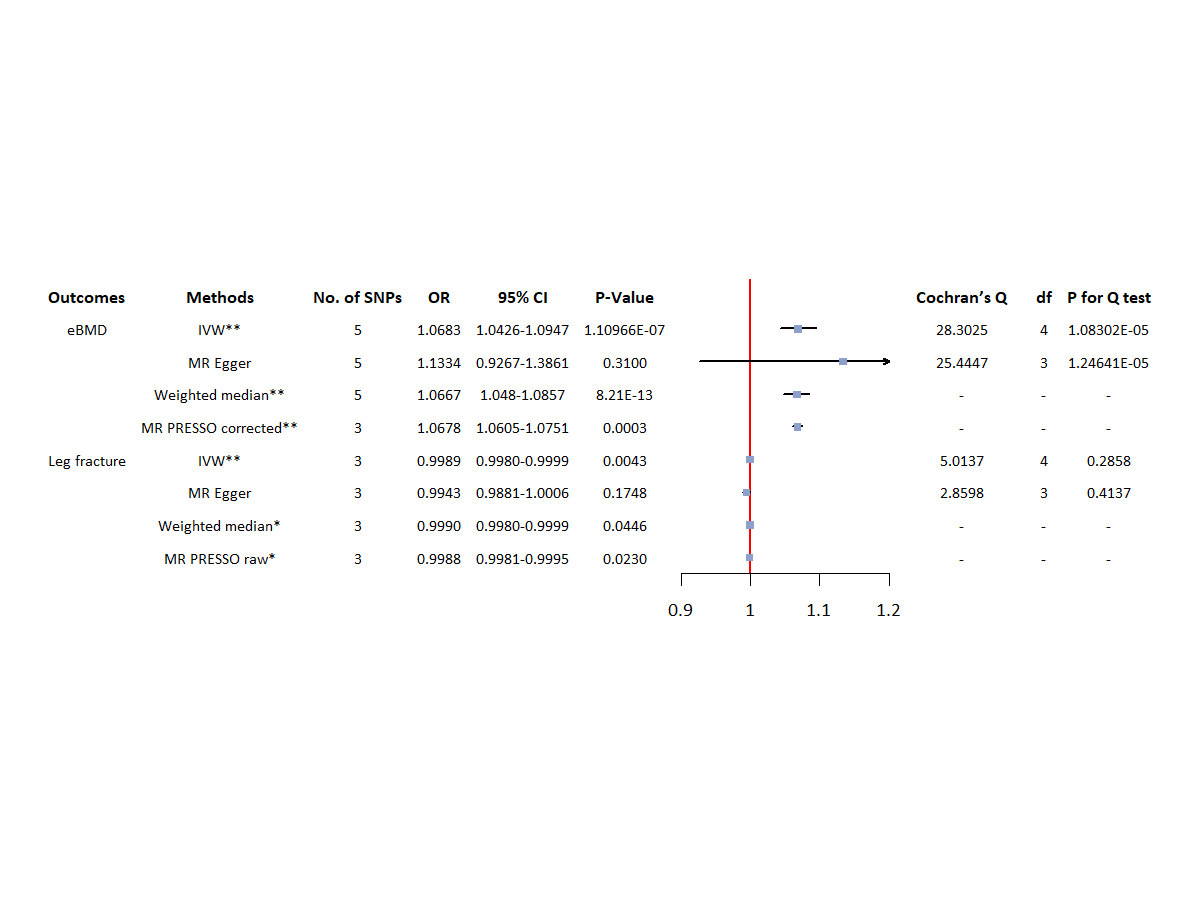


**Supplementary Figure 4**. Forest plot of the heterogeneity across all IVs (Cochran’s Q test) and analyses of childhood obesity associated SNPs potential impacts on BMD and the risk of fracture in adulthood. BMD: bone mineral density; IV: instrumental variable.
