# Supplementary material for: The causal impact of childhood obesity on bone mineral density and fracture in adulthood: A two-sample Mendelian randomization study
Source: Front Nutr. 2022 Sep 14;9:945125. doi: 10.3389/fnut.2022.945125 (PMC9515586; doi:10.3389/fnut.2022.945125)
Supplement: Supplementary file 1 [file Data_Sheet_1.zip › Supplementary Table.docx]

# Supplementary Table

Supplementary Table 1 Characteristics of 5 SNPs loci included in the MR analysis.

| **SNP** | **Chr** | **pos** | **EA** | **OA** | **EAF** | **N** | **R^2^** | ***F*** | **Exposure** | | |  | **Outcome** | | | **Outcomes** |
| --- | --- | --- | --- | --- | --- | --- | --- | --- | --- | --- | --- | --- | --- | --- | --- | --- |
|  |  |  |  |  |  |  |  |  | **beta** | **se** | ***P-value*** |  | **beta** | **se** | ***P-value*** |  |
| rs1040070 | 1 | 74977870 | C | G | 0.585 | 13848 | 0.0108 | 151.2919 | -0.1492 | 0.0269 | 2.77E-08 |  | -0.0087 | 0.0019 | 3.00E-05 | Heel bone mineral density |
| rs4854344 | 2 | 638144 | T | G | 0.837 | 13848 | 0.0163 | 229.1363 | 0.2445 | 0.0351 | 3.22E-12 |  | 0.0150 | 0.0024 | 3.90E-08 | Heel bone mineral density |
| rs571312 | 18 | 57839769 | A | C | 0.283 | 13848 | 0.0160 | 225.2299 | 0.1986 | 0.0309 | 1.25E-10 |  | 0.0146 | 0.0022 | 3.00E-07 | Heel bone mineral density |
| rs6752378 | 2 | 25150116 | A | C | 0.500 | 13848 | 0.0144 | 201.7984 | 0.1695 | 0.0262 | 1.05E-10 |  | 0.0037 | 0.0018 | 7.80E-02 | Heel bone mineral density |
| rs7138803 | 12 | 50247468 | A | G | 0.442 | 13848 | 0.0138 | 193.6038 | 0.1672 | 0.0271 | 6.50E-10 |  | 0.0116 | 0.0019 | 9.20E-07 | Heel bone mineral density |
| rs9941349 | 16 | 53825488 | T | C | 0.466 | 13848 | 0.0195 | 274.9629 | 0.1978 | 0.0267 | 1.16E-13 |  | 0.0192 | 0.0019 | 1.50E-19 | Heel bone mineral density |
| rs4854344 | 2 | 638144 | T | G | 0.837 | 13848 | 0.0163 | 229.1363 | 0.2445 | 0.0351 | 3.22E-12 |  | 0.0150 | 0.0024 | 3.90E-08 | Forearm bone mineral density |
| rs571312 | 18 | 57839769 | A | C | 0.283 | 13848 | 0.0160 | 225.2299 | 0.1986 | 0.0309 | 1.25E-10 |  | -0.0001 | 0.0002 | 6.70E-01 | Forearm bone mineral density |
| rs6752378 | 2 | 25150116 | A | C | 0.500 | 13848 | 0.0144 | 201.7984 | 0.1695 | 0.0262 | 1.05E-10 |  | -0.0001 | 0.0001 | 4.30E-01 | Forearm bone mineral density |
| rs7138803 | 12 | 50247468 | A | G | 0.442 | 13848 | 0.0138 | 193.6038 | 0.1672 | 0.0271 | 6.50E-10 |  | -0.0004 | 0.0001 | 6.70E-03 | Forearm bone mineral density |
| rs9941349 | 16 | 53825488 | T | C | 0.466 | 13848 | 0.0195 | 274.9629 | 0.1978 | 0.0267 | 1.16E-13 |  | -0.0003 | 0.0001 | 2.80E-02 | Forearm bone mineral density |
| rs9941349 | 16 | 53825488 | T | C | 0.466 | 13848 | 0.0195 | 274.9629 | 0.1978 | 0.0267 | 1.16E-13 |  | -0.0003 | 0.0001 | 2.80E-02 | Femoral neck bone mineral density |
| rs6752378 | 2 | 25150116 | A | C | 0.500 | 13848 | 0.0144 | 201.7984 | 0.1695 | 0.0262 | 1.05E-10 |  | -0.0001 | 0.0001 | 4.30E-01 | Femoral neck bone mineral density |
| rs7138803 | 12 | 50247468 | A | G | 0.442 | 13848 | 0.0138 | 193.6038 | 0.1672 | 0.0271 | 6.50E-10 |  | -0.0004 | 0.0001 | 6.70E-03 | Femoral neck bone mineral density |
| rs4854344 | 2 | 638144 | T | G | 0.837 | 13848 | 0.0163 | 229.1363 | 0.2445 | 0.0351 | 3.22E-12 |  | 0.0150 | 0.0024 | 3.90E-08 | Femoral neck bone mineral density |
| rs571312 | 18 | 57839769 | A | C | 0.283 | 13848 | 0.0160 | 225.2299 | 0.1986 | 0.0309 | 1.25E-10 |  | -0.0001 | 0.0002 | 6.70E-01 | Femoral neck bone mineral density |
| rs6752378 | 2 | 25150116 | A | C | 0.500 | 13848 | 0.0144 | 201.7984 | 0.1695 | 0.0262 | 1.05E-10 |  | -0.0001 | 0.0001 | 4.30E-01 | Lumbar spine bone mineral density |
| rs4854344 | 2 | 638144 | T | G | 0.837 | 13848 | 0.0163 | 229.1363 | 0.2445 | 0.0351 | 3.22E-12 |  | 0.0150 | 0.0024 | 3.90E-08 | Lumbar spine bone mineral density |
| rs9941349 | 16 | 53825488 | T | C | 0.466 | 13848 | 0.0195 | 274.9629 | 0.1978 | 0.0267 | 1.16E-13 |  | -0.0003 | 0.0001 | 2.80E-02 | Lumbar spine bone mineral density |
| rs7138803 | 12 | 50247468 | A | G | 0.442 | 13848 | 0.0138 | 193.6038 | 0.1672 | 0.0271 | 6.50E-10 |  | -0.0004 | 0.0001 | 6.70E-03 | Lumbar spine bone mineral density |
| rs571312 | 18 | 57839769 | A | C | 0.283 | 13848 | 0.0160 | 225.2299 | 0.1986 | 0.0309 | 1.25E-10 |  | -0.0001 | 0.0002 | 6.70E-01 | Lumbar spine bone mineral density |
| rs4854344 | 2 | 638144 | T | G | 0.837 | 13848 | 0.0163 | 229.1363 | 0.2445 | 0.0351 | 3.22E-12 |  | 0.0150 | 0.0024 | 3.90E-08 | Fractured bone site(s): Leg |
| rs6752378 | 2 | 25150116 | A | C | 0.500 | 13848 | 0.0144 | 201.7984 | 0.1695 | 0.0262 | 1.05E-10 |  | -0.0001 | 0.0001 | 4.30E-01 | Fractured bone site(s): Leg |
| rs9941349 | 16 | 53825488 | T | C | 0.466 | 13848 | 0.0195 | 274.9629 | 0.1978 | 0.0267 | 1.16E-13 |  | -0.0003 | 0.0001 | 2.80E-02 | Fractured bone site(s): Leg |
| rs571312 | 18 | 57839769 | A | C | 0.283 | 13848 | 0.0160 | 225.2299 | 0.1986 | 0.0309 | 1.25E-10 |  | -0.0001 | 0.0002 | 6.70E-01 | Fractured bone site(s): Leg |
| rs7138803 | 12 | 50247468 | A | G | 0.442 | 13848 | 0.0138 | 193.6038 | 0.1672 | 0.0271 | 6.50E-10 |  | -0.0004 | 0.0001 | 6.70E-03 | Fractured bone site(s): Leg |
| rs7138803 | 12 | 50247468 | A | G | 0.442 | 13848 | 0.0138 | 193.6038 | 0.1672 | 0.0271 | 6.50E-10 |  | -0.0004 | 0.0001 | 6.70E-03 | Fractured bone site(s): Spine |
| rs6752378 | 2 | 25150116 | A | C | 0.500 | 13848 | 0.0144 | 201.7984 | 0.1695 | 0.0262 | 1.05E-10 |  | -0.0001 | 0.0001 | 4.30E-01 | Fractured bone site(s): Spine |
| rs9941349 | 16 | 53825488 | T | C | 0.466 | 13848 | 0.0195 | 274.9629 | 0.1978 | 0.0267 | 1.16E-13 |  | -0.0003 | 0.0001 | 2.80E-02 | Fractured bone site(s): Spine |

chr: chromosome; pos: position; EA: effect allele; OA: other allele; EAF: effect allele frequency; N: sample size
